# Comparative Study of Plastomes in *Solanum tuberosum* with Different Cytoplasm Types

**DOI:** 10.3390/plants12233995

**Published:** 2023-11-28

**Authors:** Svetlana Goryunova, Anastasia Sivolapova, Oksana Polivanova, Evgeniia Sotnikova, Alexey Meleshin, Natalia Gaitova, Anna Egorova, Anatoly Semenov, Ekaterina Gins, Alina Koroleva, Evgeny Moskalev, Elena Oves, Oleg Kazakov, Aleksey Troitsky, Denis Goryunov

**Affiliations:** 1 Russian Potato Research Centre, 140051 Kraskovo, Russia; asivolapova@yahoo.com (A.S.); polivanovaoks@gmail.com (O.P.); a-mela@mail.ru (A.M.); gaitova_na@mail.ru (N.G.); anna.ivanova1995@gmail.com (A.E.); 1042180105@rudn.ru (A.S.); katya.888888@yandex.ru (E.G.); alkoroleva18@mail.ru (A.K.); kmlgwork@mail.ru (E.M.); e_oves@bk.ru (E.O.); kazakov-og@yandex.ru (O.K.); 2 National Medical Research Center for Therapy and Preventive Medicine of the Ministry of Healthcare of the Russian Federation, Petroverigsky per.10, Bld. 3, 101990 Moscow, Russia; sotnikova.evgeniya@gmail.com; 3 Belozersky Institute of Physico-Chemical Biology, Lomonosov Moscow State University, 119992 Moscow, Russia

**Keywords:** *Solanum tuberosum*, potato, cytoplasm type, chloroplast genome, phylogeny, adaptive evolution, diversity, divergence time, *rbcL*, Rubisco

## Abstract

The potato is one of the most important food crops in the world. Improving the efficiency of potato breeding is of great importance for solving the global food problem. Today, researchers distinguish between six potato cytoplasm types: A, M, P, T, W, D. In the current study, the complete chloroplast genomes of *Solanum tuberosum* accessions with five out of the six major cytoplasmic genome types were sequenced (T-, W-, D-, A-, and P-genomes). A comparative analysis of the plastomes in potato accessions with different cytoplasm types was carried out for the first time. The time of origin of the different cytoplasm types was estimated. The presence of two main groups of chloroplast genomes among cultivated potato was confirmed. Based on the phylogenetic analysis of the complete plastome sequences, five main evolutionary branches of chloroplast genomes can be distinguished within the Petota section. Samples with A- and P- cytoplasm formed isolated and distant groups within a large and polymorphic group of samples with M-type cytoplasm, suggesting that A and P genomes arose independently. The findings suggest that the diversity of the T-genome in *S. tuberosum* Group Tuberosum could be initially low due to a bottle neck already existing at the origin of the Chilean clade. Differences in the *rbcL* gene sequence may be one of the factors causing differences in economically important traits in species with A and T-type cytoplasm. The data obtained will contribute to the development of methods for molecular marking of cytoplasm types and increase knowledge about the evolution and diversity of potato.

## 1. Introduction

The potato is one of the most important food crops in the world. Improving the efficiency of the potato breeding process is of great importance for solving the global food problem. Today, researchers distinguish between six potato cytoplasm types: A, M, P, T, W, D. Three of these types (T, W, and D) are associated with the trait of male sterility in certain hybrid combinations [1]. Currently, the study of cytoplasmic genomes in potato has gained increasing attention, which is primarily due to the development of a new technology of potato breeding and seed production based on obtaining heterotic F1 hybrids by crossing inbred diploid lines [2,3].

The A-type cytoplasm is most common for *S. tuberosum* ssp. *andigena* (*S. tuberosum* group Andigena). The potato was first introduced from South America into Spain in the second half of the 16th century and by the end of the 18th and beginning of the 19th centuries had already been widely distributed all over Europe [4,5]. It is believed that it was the Andean potato that was originally introduced into Europe. The results of the analysis of herbarium specimens showed that there were no potato samples with the T-type cytoplasm, typical for Chilean origin specimens, in Europe before the beginning of the 19th century [4]. However, due to the fact that only one locus of the chloroplast genome that marks the deletion specific to the T-genome was used for the analysis, it is not known exactly what type of cytoplasmic genome the analyzed samples possessed. The A-type cytoplasm is quite rare in modern cultivars. In 2015, R. Sanetomo and C. Gebhardt identified only five out of 694 analyzed European cultivars with A-type cytoplasm in their research [6]. Interestingly, two of them (La Ratte and Pink Fir Apple) turned out to be old French varieties, bred in the middle and second half of the 19th century. Furthermore, there are two additional records that an old UK variety “Myatt’s Ashleaf”, which is one of the first European potato and another relic variety introduced into Japan by Dutch traders in the early seventeenth century, both have A-type chloroplast DNA (cpDNA) [7]. These facts suggest a greater prevalence of exactly A-type cytoplasm in the potato gene pool at the early stage of European breeding.

Among Russian and Japanese cultivars, no specimens with A-type cytoplasm were found [1,8], among Indian cultivars, only a few specimens with A-type cytoplasm were noted [9,10,11].

P-type cytoplasm was derived from *S. phureja* (*S. tuberosum* group Phureja) [12]. At present, P-type cytoplasm is very rare in the gene pool of modern cultivars. No varieties of European, Russian, or Indian breeding have been identified with this cytoplasm type [6,8,9,10,11]. However, among the 84 Japanese varieties analyzed, five varieties with P-type cytoplasm were identified, representing 6% of the selection [1].

The M-type cytoplasm is ancestral to P- and A-type cytoplasm, which are both subtypes within M-type cytoplasm [1]. Researchers identified this type of cytoplasm in wild and cultivated potato species (*S. megistacrolobum* Bitt., *S. raphanifolium* Ca’rd. et Hawkes, *S. acroscopicum* Ochoa, *S. bukasovii* Juz., *S. canasense* Hawkes, *S. candolleanum* Berth., *S. coelestipetalum* Vargas, *S. dolichocremastrum* Bitt., *S. leptophyes* Bitt., *S. marinasense* Vargas, *S. medians* Bitt., *S. multidissectum* Hawkes, *S. multiinterruptum* Bitt., *S. ajanhuiri* Juz. et Buk., *S. juzepczukii* Buk., *S. tuberosum* L. ssp. *andigena* Hawkes, *S. acaule* Bitt.).

M-type cytoplasm appears to be the rarest in the modern gene pool. Single examples with this genome type have been identified among samples of breeding material, but not a single sample with this cytoplasm type has been identified among European, Russian, Japanese, and Indian varieties [1,6,8,9,10,11]. A study by R. Sanetomo and C. Gebhardt showed a correlation with quantitative resistance to late blight for M-type cytoplasm. It has been suggested that *S. acaule* might be the most possible donor of this cytoplasm type for the samples identified in this study [6].

The most common cytoplasm type in modern potato varieties is the “Chilean”, or T-type, typical for *S. tuberosum.* ssp. *tuberosum* (*S. tuberosum* cultivars (group Tuberosum) and their Chilean landrace progenitors (group Chilotanum). According to M. Ames and D. Spooner, the first European sample of a potato with T-type cytoplasm has been dated back to 1811, and all samples of the selection that were herbarized at the beginning of the 20th century had the Chilean T-type of cpDNA [4]. The expansion of this type of cytoplasm throughout the 19th century was facilitated both by the disappearance of much of the gene pool of old European varieties during the *Phytophthora* epidemic in the mid-1840s and by the features specific to varieties with the T-genome. First of all, samples with this type of cytoplasm have a set of economically useful traits, which were supported by selection, leading to an increase in the occurrence of this type of cytoplasm in the gene pool of modern varieties. Samples originating from Chile had the best ability to form tubers at the latitude of Europe [13]. In classical studies using reciprocal hybrids, it has been shown that the cytoplasmic genome of *S. tuberosum* ssp. *tuberosum* (mostly T-type cytoplasm) is characterized by a higher percentage of tuberization, higher tuber yield, higher tuber numbers, and earlier vine maturity compared with that of *S. tuberosum* ssp. *andigena* (mostly A-type cytoplasm) [6,14,15,16,17,18]. In addition, this type of cytoplasm is associated with male sterility, expressed as various abnormalities in the development of reproductive organs and as the plant’s inability to set berries [8,19]. Due to this feature, samples with this type of cytoplasm were predominantly used as the maternal form, leading to a further spread of T-type cytoplasm in the gene pool [19,20]. In the 20th century, wild potato species began to be intensively included in breeding, primarily as donors of resistance to pathogens, and the frequency of occurrence of the T-type decreased. Currently, the frequency of this cytoplasm type among cultivars is 69.2% for European cultivars, 40.0% for Russian and surrounding countries, 73.8% for Japanese cultivars, and from 77.2% to 86.8% for Indian cultivars [1,6,8].

In the 20th century, wild species began to be actively involved in potato breeding in order to introgress genes of agronomic importance, such as resistance to pathogens, which increased the diversity of cytoplasm types. The D-type cytoplasm was introduced into the potato gene pool by crosses with the hexaploid wild Mexican species *Solanum demissum* Lindl., which has often been used in breeding as a source of resistance to the most serious disease, late blight (*Phytophthora infestans*) [21,22,23].

Potato samples with the D-type of cytoplasm are characterized by the functional sterility of pollen. In this case, plants produce morphologically normal, well-stainable, but non-functional pollen grains [1,24]. Currently, this type of cytoplasm is very common. Among 694 European cultivars, 148 with D-type of cytoplasm (21.3%) were identified [6]. Among Japanese varieties, the percentage of varieties with this genome type was 20.2% [1].

Among Indian cultivars, the frequency of D-type of cytoplasm was 16.36% according to G. Vanishre et al. [9] and 19.3% according to S. Sood et al. [10]. The highest frequency of D-type of cytoplasm was in bred cultivars in Russia and adjacent countries—50.8% [8].

The W/γ subtype of cytoplasm, associated with the tetrad form of male sterility, has been introduced into the modern potato gene pool from *S. stoloniferum* species along with the potato Y virus resistance gene *Rysto* [6,25,26].

Among the varieties of European selection, the frequency of this type of cytoplasm is 6.5%; among the varieties of Russia and adjacent countries—8.7% [6,8]. It is interesting that the W type of cytoplasm was not detected in Japanese varieties, and in Indian cultivars, only one sample with this type of cytoplasm was identified [1,10].

To identify cytoplasm types in potato, a set of five molecular markers (A, T, SAC, D, and S) developed by K. Hosaka and R. Sanetomo [1] is currently the most widely used.

The T marker was designed by K. Hosaka [27] to detect a previously identified 241-bp long deletion in the *trnV/ndhC* inter-genic region of the cpDNA specific to *S. tuberosum* ssp. *tuberosum* (T-type cytoplasm). This deletion was detected through an analysis of restriction fragment patterns of cpDNA, and information about the flanking sequences was obtained through cloning and further sequencing of restriction fragments [28,29]. The S marker is the SSR marker NTCP6 in the *rps16/trnQ* intergenic region, developed based on the complete sequence of the tobacco chloroplast genome [30,31]. Marker D was developed from the *S. demissum*-derived cytoplasm-specific Band 1 flanking sequence, which was previously identified as a 170 bp EcoRI and MspI (or HpaII) double-digested DNA fragment [32]. It was previously shown that this fragment was maternally inherited from *S. demissum,* but the intracellular origin as to whether it is a part of cpDNA or mtDNA remained unknown [32]. The CAPS markers SAC and A were developed by K. Hosaka and R. Sanetomo [1] based on a comparison of the BamHI restriction fragment patterns of cpDNA with data from the complete sequence of potato cpDNA [33]. As a result, primers flanking the diagnostic BamHI recognition sites were developed [1].

The cytoplasmic genome includes both mitochondrial and chloroplast genomes which are inherited jointly through the maternal line. However, it is the chloroplast genome that is most commonly used in the evolutionary studies of angiosperms due to its non-recombinant nature. In addition, four out of five molecular markers applied for cytoplasm type determination in potato have plastome localization. That is why we focused on investigating plastomes associated with various potato cytoplasm types.

So far, cytoplasmic genome types for more than a thousand potato samples have been established and several hundreds of complete chloroplast genomes of the genus *Solanum* have been sequenced, including a large-scale analysis of 202 plastid genomes of wild and cultivated diploid potatoes, *Solanum* section Petota, performed by B. Huang et al. [34]. Nevertheless, no comparative analysis of the complete sequences of potato plastomes with different cytoplasmic types has been performed so far.

In the current study, the complete chloroplast genomes of six *Solanum tuberosum* accessions with five out of the six major cytoplasmic genome types were sequenced (T-, W-, D-, A-, and P-genomes). The data obtained will contribute to the development of methods for the molecular marking of cytoplasm types and increase knowledge about the evolution and diversity of potato.

## 2. Results

### 2.1. Structural Characteristics of Plastid Genomes of Solanum tuberosum Acessions

The chloroplast genomes of six samples of *Solanum tuberosum* with different cytoplasmic types (A, P, T, D, W) were sequenced and assembled into circular molecules (Figure S1). The average genome coverage varied from 531× to 804× (Table 1). GC-content was 37.9% for all sequences. The size of the plastomes varied from 155,296 bp for the sample with T-type cytoplasm to 155,562 bp for the sample with D-type cytoplasm. The length difference in samples with A-type cytoplasm was only 1 bp. The length of the large single-copy (LSC) region ranged from 85,737 to 86,003 bp, and the length of the small single-copy (SSC) region from 18,364 to 18,376 bp (Table 1). The length of inverted repeats (IRa, IRb) in samples with T, D, A, and P types of cytoplasmic genomes was the same—25,593 bp—and in the sample with W type of cytoplasm it was one nucleotide shorter. Gene content was identical for all sequenced genomes. A total of eighty-six CDS, eight rRNA genes, and thirty-seven tRNA-genes were annotated in each genome, for a total of 133 genes including two pseudogenes (*ycf1*, *rps19*). The shortest among the protein-coding genes in all sequenced plastomes is *petN* (90 bp). The longest one is *ycf2* (6837 bp), occurring in two copies in each of the presented genomes. Only 15 protein-coding genes contain introns.

### 2.2. Repetitive Elements and SSR-Analysis

**Figure 1:**
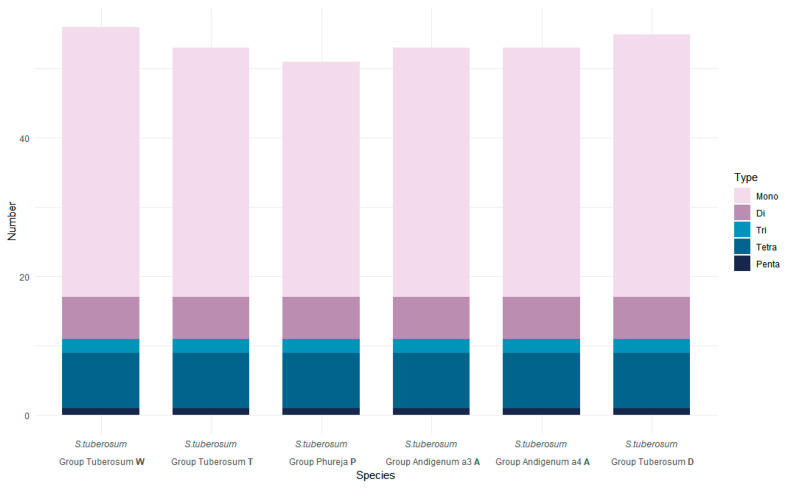
SSR-content in plastomes of *S. tuberosum* accessions. Different colors in the figure represent different types of microsatellite repeats. Occurrences of the SSRs of different types are shown on the *Y*-axis.

The number of SSR detected in the analyzed genomes ranged from 51 in *S. tuberosum* Group Phureja (P-type cytoplasm) to 56 in *S. tuberosum* Group Tuberosum cv. Nakra (W) (Table S1). The samples with T and A-type cytoplasm showed the same number of SSR repeats and was equal to 53. The total length of SSR repeats in the genome ranged from 584 bp in *S. tuberosum* Group Phureja (P-type cytoplasm) to 625 bp in *S. tuberosum* Group Tuberosum cv. Nakra (W), which was 0.376% to 0.402% of the genome size, respectively. The number of SSR repeats within protein-coding sequences was the same for all samples and was equal to six. The highest number of mononucleotide repeats was detected in *S. tuberosum* Group Tuberosum cv. Nakra (39), and the lowest in *S. tuberosum* Group Phureja (34). The number of di-, tri-, tetra-, and pentanucleotide repeats was identical in all samples and were 6, 2, 8, and 1, respectively. No hexanucleotide repeats were detected during the analysis. In general, all analyzed genomes have a similar SSR-content. The differences are explained by the variability only in the number of mononucleotide repeats (Figure 1, Table S1).

**Figure 2:**
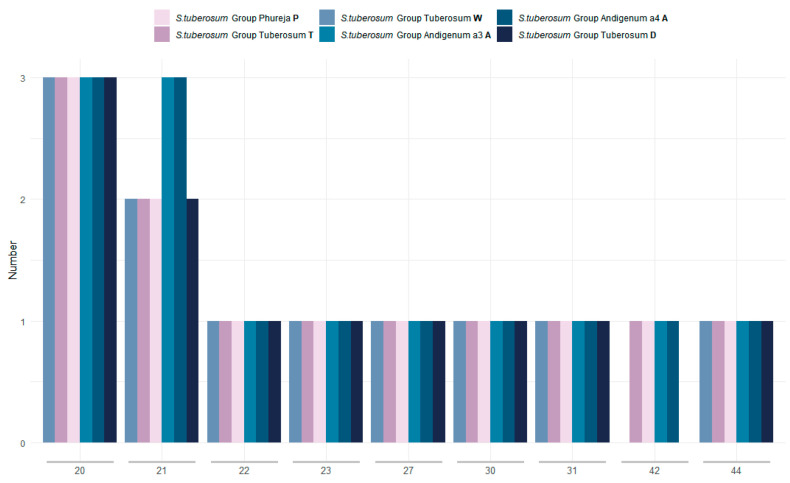
Repetitive elements of different length in plastomes of *S. tuberosum* accessions. Lengths (bp) of the repeat subunits are shown on *X*-axis.

Analysis of longer repetitive elements (minimum 20 bp) with REPuter detected between 11 (for W and D-genome types) and 13 (for A-genome type) repeats (Table S2, Figure 2). Total repeat length varied from 628 bp for *S. tuberosum* Group Tuberosum cv. Nakra (W) to 727 bp for both accessions of *S. tuberosum* Group Andigenum, which comprises 0.404% and 0. 468% of the genome length, respectively.

The number of repeats in which all subunits were located within the genes ranged from four (for W and D-types) to five repeats (in all other samples). The number of repetitive elements in which all subunits were in intergenic spacers was the same in all samples and equaled four. The repeat content is similar in all sequenced samples. At the same time, *S. tuberosum* Group Andigenum samples contain one more 21 bp repeat than the others (three vs. two in all other samples). In addition, samples with the W and D types of cytoplasm do not have a 42 bp repeat.

### 2.3. Comparison and Nucleotide Diversity Analysis of Assembled Genomes

The alignment length of the six sequenced plastomes was 155,715 bp. The minimum similarity was between genomes with P-type and T-type cytoplasm, while the pairwise similarity was high even in this case and reached 99.583%. The maximum similarity (99.999%) was revealed for two sequences with the A-type genome (Table S3).

**Figure 3:**
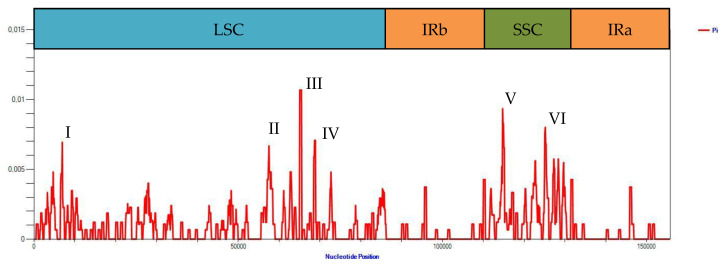
Nucleotide diversity among chloroplast genomes of six potato accessions. On the top of the figure, boundaries of the large single-copy (LSC) region, small single-copy (SSC) region, and two inverted repeat (IRa, IRb) regions are indicated. Roman numerals correspond to the six highest peaks of nucleotide diversity value.

Analysis of nucleotide diversity in the alignment of chloroplast genome of six potato accessions with different cytoplasm types showed that the unique regions of the genome were more diverse compared to IR (Figure 3). The six highest peaks can be identified on the graph, the first of which corresponds to the 6219–7598 region of the alignment and is flanked by the *rps16* and *psbK* genes (corresponds to the peak I on the Figure 3). The second peak (II) starts in the *atpB* gene and ends in the *accD* gene (positions 55,348–59,155 of the alignment), the third (III) begins in the *petA* gene and ends in the *petA*-*psbJ* intergenic spacer (64,783–65,708), and the fourth (IV) is located in the region from the *petL*-*petG* intergenic spacer to the *psaJ* gene (68,225–69,326). These four peaks are located in the LSC region. The two remaining peaks are localized in the SSC region. The fifth one (V) starts in the *ndhF* gene and ends in the *ccsA* gene (112,968–116,894); the sixth (VI) starts in the *ndhH* gene and ends in the *ycf1* gene (124,405–126,204).

### 2.4. Localization of Molecular Markers for Identification of Cytoplasm Types (A, T, SAC, D, and S) in Potato Chloroplast Genomes

The primer sequences for the molecular markers A, T, SAC, D, and S that were designed by K. Hosaka and R. Sanetomo to identify potato cytoplasm types [1] were searched in complete chloroplast genome sequences of *S. tuberosum* cv. Barin with the D type of cytoplasm. The D type of cytoplasm was selected due to the fact that only accessions with that type of cytoplasm were characterized by the presence of all the above mentioned markers, including D [1]. The results are summarized in Table 2. No primers allowing amplification specific for D-type cytoplasm were identified.

### 2.5. Potato Chloroplast Genome Phylogeny and Molecular Dating Analyses

**Figure 4:**
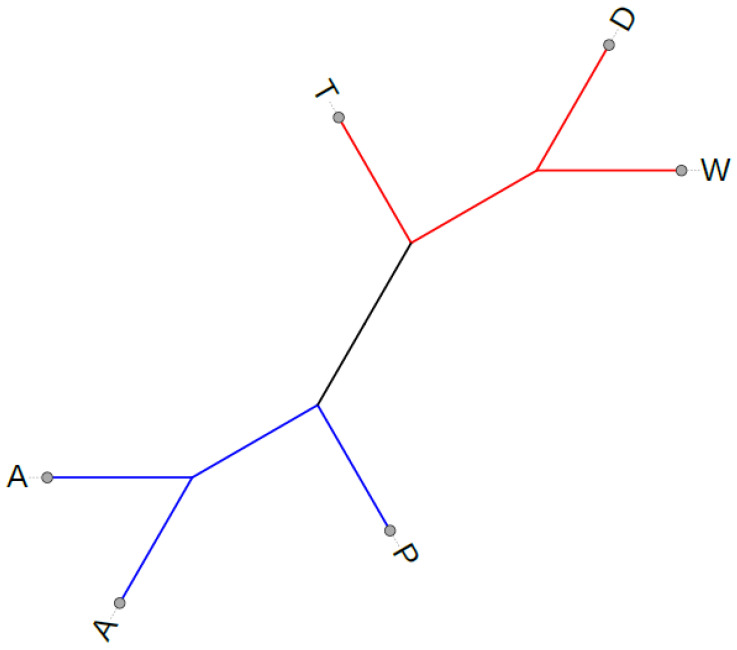
Unrooted maximum likelihood tree of six potato plastomes. Supports for all branches are maximal. The capital letters denote different cytoplasm types.

The accessions formed two main clades on the tree (Figure 4), the first including accessions with T-, D-, and W-type cytoplasm, and the second accessions with P- and A-type cytoplasm. The chloroplast genome sequences of samples with D-, W-type cytoplasm were closer to each other than to those with T-type, and samples with A-type cytoplasm were grouped together.

**Figure 5:**
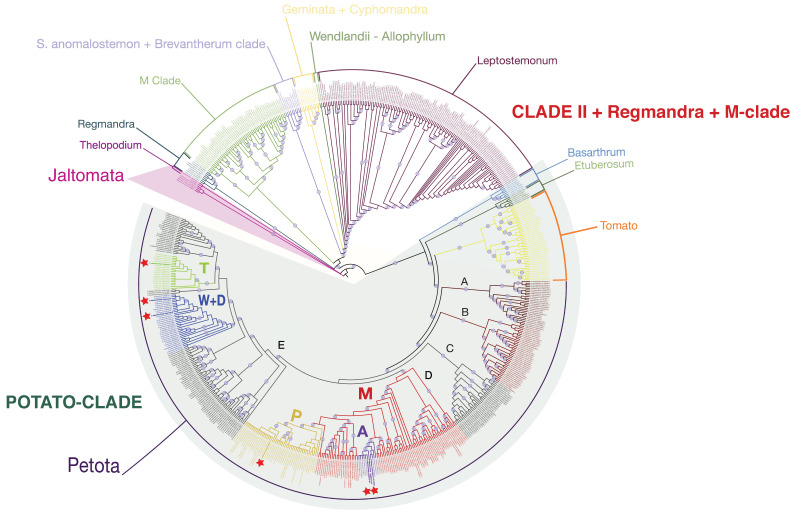
Phylogenetic tree of *Solanum* plastomes. *Jaltomata* accessions were selected as an outgroup. The nodes with support ≥85% are denoted by circles. Accessions sequenced in the current study are marked by an asterisk. The letters A–E denote five main clades within the section Petota; the bold colored letters denote different cytoplasm types.

To analyze the phylogenetic relationships of T-, D-, W-, P- and A-type plastomes, they were aligned with sequences of other *Solanum* species from the GenBank database. All plastome sequences included in the analysis were searched for primer sequences used to determine cytoplasmic type and thus identified sequences belonging to M, A, P, T, and W + D types. On the tree constructed using over 600 sequences from the GenBank database, samples with T-, D-, W-type cytoplasm and samples with P- and A-type cytoplasm also fall into different clades (Figure 5, Figure S2).

Overall, two main clades can be distinguished on the tree (Figure 5). The first (Potato clade according to B. Gagnon et al. [35]) includes species of sections Petota, Lycopersicon (Tomato), Etuberosum, and Basarthrum. The second clade includes all remaining species of the genus *Solanum*. The only exception is *S. dimorphandrum*, which is isolated from all of the others and is grouped together with representatives of the genus *Jaltomata* (Figure S2).

Within the section Petota, five main branches can be recognized on the tree. The basal clade (clade A) includes specimens of *S. cardiophyllum*, *S. bulbocastanum*, *S. polyadenium*, *S. stenophyllidium*, *S. bukasovii*, *S. jamesii*, and *S. pinnatisectum*.

The second (clade B) consists of species *S. albornozii*, *S. andreanum*, *S. humectophilum*, *S. chomatophilum*, *S. chiquidenum*, *S. cantense*, *S. acroglossum*, *S. hypacrarthrum*, *S. multiinterruptum*, *S. mochiquense*, *S. blanco-galdosii*, *S. sogarandinum*, *S. cajamarquense*, *S. paucissectum*, *S. piurae*.

The third clade (clade C) contains accessions of *S. gourlayi*, *S. incamayoense*, *S. microdontum*, *S. brevicaule*, *S. sparsipilum*, *S. canasense*, and *S. leptophyes*.

The fourth clade (D) comprises accessions with the M-, A-, and P-cytoplasm types. Among the species with M- cytoplasm were samples of species *S. ambosinum*, *S. raphanifolium*, *S. lignicaule*, *S. bukasovii S. multidissectum*, *S. bukasovii*, *S. tacnaense*, *S. acaule*, *S. x juzepczukii*, *S. megistacrolobum*, *S. boliviense*, *S. cajamarquense*, *S. ahanhuiri*, *S. infundibuliforme*, *S. gracilifrons*, *S. tuberosum* subsp. *andigenum*, *S. tacnaense*, *S. canasense*, *S. achacachense*, *S. medians*, *S. multiinterruptum*, *S. tarapatanum*, *S. marinasense*, *S. limbaniense*, *S. leptophyes*, *S. candolleanum*. Samples with A- and P- cytoplasm formed isolated and distant groups within a large and polymorphic group of samples with M-type cytoplasm. The A-genome group contains representatives of *S. bukasovii*, *S. stenotomum*, *S. chaucha*, and *S. tuberosum* subsp. *andigenum* (*S. tuberosum* Group Andigenum), including two samples sequenced in this study. The P-genome group comprises samples of *S. abancayense*, *S. stenotomum* subsp. *goniocalyx*, *S. phureja* (*S. tuberosum* Group Phureja), *S. stenotomum*, *S. curtilobum*, *S. tuberosum*, *S. tuberosum* subsp. *andigenum*, *S. ambosinum*, *S. canasense*, *S. marinasense*, *S. tuberosum*, *S. stenotomum* subsp. *stenotomum*, and *S. bukasovii*.

The fifth clade (E) consists of several groups, including a group of samples with a T- and a group of samples with D- and W-type cytoplasm. Due to the fact that there is no marker in the chloroplast genome sequence that allows us to distinguish between the D- and W- types of cytoplasm, it is impossible to identify D- and W- clades separately within this cluster. In this case, samples with D- and W-type cytoplasm fall into one group, which includes other *S. tuberosum* samples, samples of *S. demissum*, which is a donor of D-type cytoplasm, a sample of *S. stoloniferum*, which is a donor of W-type cytoplasm, and a sample of *S. hougasii*. The other branches of the D/W clade include accessions of the species *S. verrucosum*, *S. iopetalum*, *S. hjertingii*, *S. brevicaule*, *S. gourlayi*, *S. hondelmannii*, and *S. avilesii*.

The group of accessions with T-type cytoplasm includes representatives of the *S. tuberosum*, *S. tarijense* species, and one specimen of *S. berthaultii*.

A separate branch within the E-clade is formed by accessions of *S. tarijense* with a cytoplasm different from T-type together with accessions of *S. avilesii*, *S. gourlayi*, *S. berthaultii*, *S. hondelmannii*, *S. boliviense*, *S. sparsipilum*, and *S. laxissimum*. One accession of *S. violaceimarmoratum* is also in this group. The other three accessions of this species form an isolated branch within the E-clade.

The remaining subclades within the E-clade include samples of *S. kurtzianum*, *S. vernei*, *S. spegazzinii*, *S. buesii*, *S. laxissimum*, *S. gandarillasii*, *S. chacoense*, *S. microdontum*, *S. okadae*, *S. venturii*, *S. brevicaule*, *S. pampasense*, *S. velardei*, and *S. commersonii*, and one sample of *S. tarijense* MH021574-1.

**Figure 6:**
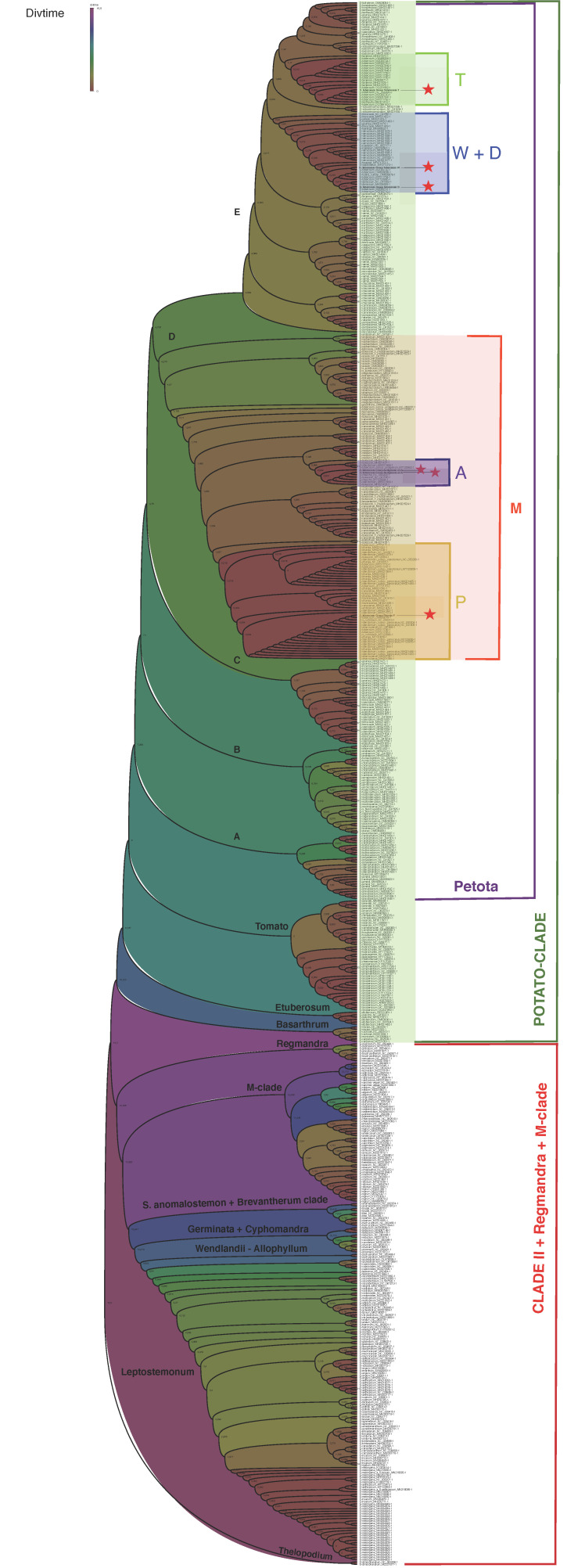
Dated tree of *Solanum* plastomes. Accessions sequenced in the current study were marked by asterisk. Colors reflect the difference in divergence time.

The constructed tree has been calibrated based on the previously obtained minimum age estimates for major splits within the Solanaceae family [36]. According to this analysis, the divergence time of the Petota section (split A clade/(B + C + D + E) clade) is estimated to be 7.29 MYA (CI95 = 7.1–8.0), the branching time of clade A is 6.29 MYA (5.55–7.13), and the branching time of clade B is 6.54 MYA (6.38–6.75). The split between clade C and the (D + E)-clade is 4.76 MYA (3.97–5.70), the time of divergence within clade C is 1.17 MYA (0.74–1.85), the split between clade D and clade E is 4.71 MYA (3.78–5.70), the branching time of clade D (genome M) is 4.28 MYA (3.25–5.62), and the divergence of clade E is 2.66 MYA (1.68–4.23) (Figure 6).

Within clade D, the split between the A genome and the nearest cluster of samples with the M genome (comprising *S. medians* samples MH021512-1, NC 041618-1, MH021514-1, MH021515-1, MH021513-1, MH021516-1) is 1.50 (0.56–3.61); divergence within the group of samples with A-genome is estimated to be 1.0 MYA (0.31–3.19). At the same time, the divergence time of the group of samples including representatives of *S. tuberosum* species with A-type genome is only 0.03 MYA (0.01–0.10). The split between the P genome and the closest cluster of samples with the M genome (*S. bukasovii* MH021435-1+ *S. leptophyes* MH021506-1) is 1.26 MYA (0.49–3.39); divergence time within the group of samples with P-genome is 0.02 MYA (0.0004–0.604). The time to the most recent common ancestor (MRCA) of P and A-genome groups is 1.50 MYA (0.60–3.61).

Within the E clade, the divergence time between the group of samples including clusters of samples with T and W + D genomes and the nearest group of samples of the *S. kurtzianum*, *S. vernei*, *S. spegazzinii*, *S. buesii*, *S. laxissimum*, *S. tarijense*, and *S. gandarillasii* species is 2.22 MYA (1.27–3.87). The time of existence of MRCA of the group of samples with W + D genomes and the group of samples including the T-genome clade is 2.03 MYA (1.08–3.82). The branching time of the group with W + D genomes is 0.94 MYA (0.40–2.23). The split between the group of samples including samples of *S. tuberosum*, *S. demissum*, *S. stoloniferum,* and other samples with W/D-type genomes is 0.25 MYA (0.06–0.94).

The divergence time of the group of samples with the T-genome from the nearest cluster of samples of the *S. tarijense*, *S. gourlayi*, *S. avilesii*, *S. berthaultii*, *S. hondelmannii*, *S. boliviense*, *S. sparsipilum*, *S. laxissimum*, and *S. violaceimarmoratum* species with a different cytoplasm type is 1.42 MYA (0.66–3.08). The branching time of the cluster of samples with T-genome is 1.17 MYA (0.48–3.08). Meanwhile, the divergence time of the group of samples including representatives of *S. tuberosum* species with T-type genome is only 0.01 MYA (0.002–0.087).

### 2.6. Polymorphism of Protein Sequences Encoded by Potato Chloroplast Genomes

It is known that potato accessions with T-type cytoplasm outperform those with A-type cytoplasm by a whole complex of economically useful traits. At the same time, the fact that this was confirmed in reciprocal crosses [6,14,15,16,17,18] indicates the contribution of genes from the cytoplasmic genome to the control of these traits, rather than the different frequency of allelic variants of the nuclear genome in samples with different cytoplasm types. Therefore, it was of interest to investigate the differences in the coding sequences of chloroplast genomes in samples with different cytoplasm types. In this regard, the polymorphism of amino acid sequences of proteins encoded by plastome genes in six *S. tuberosum* samples sequenced in the present study was analyzed. As a result, polymorphism was detected for only 20 genes. Among these genes, differences between A and T genome types were detected for only 15 genes, and differences between (A + P) and (T + W + D) genome groups were shown for 13 genes. Among these 15 genes were those that encode ATP synthase, Maturase K, NADH dehydrogenase, Photosystem II, Rubisco Large subunit, DNA dependent RNA polymerase, Large and Small subunits of ribosome, and *ycf1*. The largest total number of substitutions and the number of substitutions distinguishing A and T genome types were found in *rbcL* and *ycf1* genes (Table 3).

For all 20 genes for which variability in their protein sequences was detected, the Ka/Ks ratio was analyzed in the sequenced plastomes. Positive selection (adaptive evolution) and a Ka/Ks ratio > 1 were observed only for *rbcL* (Table 4). Meanwhile, for the genus *Solanum* as a whole (646 samples), positive selection for the *rbcL* gene was not detected and the Ka/Ks ratio was 0.7628.

In this regard, amino acid substitutions in the protein sequences of the large subunit of ribulose 1,5-bisphosphate carboxylase/oxygenase (Rubisco), encoded by the *rbcL* gene, between the genome groups (A + P + M) and (T + W + D) were analyzed in more detail. For this purpose, an alignment of the amino acid sequences of *rbcL* gene was carried out for six samples sequenced in this study, those from the M-type chloroplast genome from the database (*S. ambosinum* voucher PI 365,317 plastid, complete genome MH021403.1), and the spinach (NP_054944.1). For spinach Rubisco, the structure of the enzyme complex was previously determined based on crystallographic analysis. As a result, the structure of the complex has been clarified and a number of conserved interaction areas that may be of functional significance have been identified [37]. The alignment showed a high sequence conservation of the Rubisco large subunit in spinach and potato. The pairwise identity between protein sequences of spinach and *S. tuberosum* with M, A and P plastomes was 93.263%, and between spinach and *S. tuberosum* with T, D and T plastomes it was 93.684%. No indels were revealed in the alignment, except insertions of two amino acids on C-terminus of *Solanum* sequences. This provided an opportunity to compare the positions of amino acid residues in potato and spinach proteins and to determine which structural and functional regions of the protein the identified variable positions belong to.

In the amino acid sequences of Rubisco large subunits, seven amino acid substitutions were identified between the accessions with M, A, and P types of cytoplasm from the one side and with T, W, and D types of cytoplasm on the other side (Table 5). Two substitutions (positions 117 and 142) are located in the N-terminal domain, the others are located in the C-terminal domain. In position 309 a hydrophobic non-polar aliphatic amino acid is replaced by another one; in position 117 a hydrophobic non-polar aromatic amino acid is changed to a hydrophobic non-polar aliphatic one; in positions 142, 230, and 449, hydrophilic polar uncharged amino acids are replaced by hydrophobic non-polar aliphatic amino acids; in position 262, on the contrary, a hydrophobic non-polar aliphatic amino acid is replaced by a hydrophilic polar uncharged one; and in position 439, a hydrophilic polar uncharged amino acid is replaced with a hydrophilic polar basic one.

## 3. Discussion

In the present study, the complete chloroplast genomes of *S. tuberosum* accessions with different cytoplasm types were sequenced. The primer sequences for markers A, T, SAC, and S were localized to the chloroplast genome sequence (Table 5). However, no primers allowing amplification of a cytoplasm-specific D-type DNA fragment (D-marker) were identified. The D marker was designed by R. Sanetomo and K. Hosaka in 2011, based on the sequence flanking the 170 bp long fragment “Band1” identified by restriction analysis. This fragment was shown to be maternally inherited from *S. demissum*, but it could not be determined whether this fragment was part of the mitochondrial or chloroplast genomes [32]. To clarify the intracellular localization of the fragment, the sequence of the fragment “Band1” (NCBI Genbank Accession No. HR505437) was aligned with sequences from the Genbank database using the blastn algorithm. As a result, the best matches were found with 100.00% identity, E-value 3 × 10^−81^ and query coverage 100% with the sequences of *S. tuberosum* mitochondrial genome (LC649824, LC649821, MW122984). Thus, the D marker can be considered localized in the mitochondrial genome.

The similarity level of the chloroplast genome sequences was quite high and ranged from 99.58 to 99.93% for plastome pairs of samples with different cytoplasm types. All sequences had the same set of genes, similar length, and a similar set of repetitive elements.

On the tree constructed using sequences from the GenBank database, two main clades can be distinguished, the first (Potato clade according to B. Gagnon et al. [35]) includes species of sections Petota, section Lycopersicon (Tomato), section Etuberosum, and sect. Basarthrum. The second includes all remaining species of the genus *Solanum*. Thus, the tree topology constructed in the present study based on the alignment of complete chloroplast genome sequences using the FastTree algorithm corresponds to the tree topology constructed earlier by B. Gagnon et al. [35], using the unpartitioned maximum likelihood analysis based on 160 loci representing exons, introns, and intergenic regions of plastomes of *Solanum*.

It should be noted that, at the same time, *S. dimorphandrum* is isolated from all other representatives of the genus and is grouped together with representatives of the genus *Jaltomata* (Figure S2). This may indicate the possible need to revise the boundaries of the genus *Solanum*. In addition, it should be mentioned that representatives of the Thelopodium clade, to which *S. dimorphandrum* belongs, were absent in the work of R. Olmstead et al. [39], where it was reported that *Jaltomata* is sister to *Solanum*. At the same time, long branch attraction bias cannot be excluded. Based on the performed analysis, five main evolutionary branches of chloroplast genomes (clades A–E) can be distinguished within the section Petota. The basal clade A includes specimens of predominantly North American species. The only exception is the one accession of South American species *S. bukasovii*, which is a member of this clade as well.

The mentioned clade corresponds to the 1 + 2 clade distinguished by B. Huang et al. [34]. According to the authors, this clade also includes the epiphytic species *S. morelliforme* Bitter and Muench with a disjunctive range that covers the area from central Mexico (southern Jalisco to Querétato and Veracruz) to southern Honduras, and also includes one isolated population in Bolivia [40,41].

Clade B corresponds to clade 3 identified by B. Huang et al. [34]. Clade C, distinguished in the present study, is not recognized as a separate group by B. Huang et al. [34], but is considered part of the southern subgroup of Clade 4.

Clade D combines samples with M-, A-, and P-genomes. Meanwhile, specimens with A- and P-genomes formed distant isolated groups within a large and polymorphic group of specimens with M-type cytoplasm. This clade corresponds to the clade 4 north + clade 4 cultivated isolated by B. Huang et al. [34].

Clade E contains several groups, including a cluster of samples with a T-genome and those with D- and W-type cytoplasm. B. Huang et al. joins this clade with clade C into clade 4 south [34]. Interestingly, this clade includes, along with representatives of South Américan species, accessions of several North American and Central American species: *S. demissum*, *S. stoloniferum*, *S. hougasii*, *S. verrucosum*, *S. iopetalum*, and *S. hjertingii*. Previously, B. Huang et al. have already shown that the diploid species *S. verrucosum* from Mexico groups together with southern South American species [34]. However, polyploid species were not included in this study. Thus, two of the five clades include representatives of North American and Central American potato species, suggesting two independent cases of introgression of species of section Petota into North and Central America.

The clustering of potato accessions with T-, D-, W-, P- and A-cytoplasm type on the tree was consistent with the idea that the T- and D-type cytoplasm and the P- and A-type cytoplasm are relatively distinct cytoplasmic types within the W- and M-type cytoplasm [32,42,43].

At the same time, as already mentioned, samples with the A and P types of cytoplasm form two distant from each other groups, which indicates their independent origin. Interestingly, only the diploid species *S. bukasovii* (synonymous with *S. candolleanum*) is represented in the A-genome group among wild species. The P-genomic group includes samples of wild species *S. abancayense*, *S. ambosinum*, *S. canasense*, *S. marinasense*, and *S. bukasovii*, which are also all synonymous with *S. candolleanum*. Thus, the results obtained are consistent with the data on the origin of *S. tuberosum* groups Stenotomum, Phureja, and Andigenum from the diploid species *S. candolleanum*, which is a representative of the northern clade of the *S. brevicaule* complex [44,45]. However, given the independent origin of the A- and P-type cytoplasmic genomes, it seems unlikely that Andean cultivated tetraploids (*S. tuberosum* group Andigena), characterized by a predominantly A-type cytoplasmic genome, evolved directly from early landrace diploids (*S. tuberosum* groups Stenotomum and Phureja) through autopolyploidization, as is sometimes suggested [46]. It is possible to assume either the independent domestication of representatives of the two groups, or the origin of the *S. tuberosum* group Andigenum from the crossing of a representative of the A-genomic group as a maternal form with a representative of the P-genomic group.

The clade of samples with the T-type of cytoplasmic genome included fifteen accessions of *S. tuberosum* and five accessions of the wild species *S. tarijense* and *S. berthaultii*. Although these species were initially described as independent species, evidence was subsequently obtained that it would be better to combine them into a single highly polymorphic taxon [47]. Meanwhile, seven different haplotypes of chloroplast genome were detected in the samples of this clade. Three haplotypes were detected only in *S. tuberosum*, three in *S. tarijense* accessions, and one was common to *S. tuberosum* and *S. berthaultii* (Table 6).

Previously, in a study of 566 accessions of 35 wild potato species, the “241 bp” deletion characteristic of the T-type cytoplasm was found in only fourteen accessions of *Solanum tarijense* (including *S. berthaultii* Hawkes) Hawk. and two accessions of the species *Solanum neorossii* Hawk. and Hjert. [27]. The frequency of T-type cytoplasm among *S. tarijense* (including *S. berthaultii* Hawkes) was about 18–20% [27,48,49]. *Solanum tarijense* is a diploid species distributed from central Bolivia to northwest Argentina [50]. As T-type chloroplast DNA was not found in diploid cultivated forms, it was suggested that Chilean tuberosum originated from crossing of *S. tarijense* with T-type chloroplast DNA as a female with Andigena [51]. In 2018, studies were performed to experimentally verify the proposed evolutionary pathway by synthesizing long-day adapted, edible tetraploid potatoes by crossing 10 accessions of *S. tarijense* with T-type cytoplasm used as the females and 32 Andean tetraploid landraces (Andigena) used as the males. As a result, data supported the hypothesis that the Chilean tuberosum originated by selection for long-day adaptability from tetraploid hybrids that occurred via the fertilization of a 2n egg of *S. tarijense* by n pollen of Andigena [7].

The absence of species other than *Solanum tuberosum* and *Solanum tarijense* (*S. berthaultii*) in the T clade, and the presence of identical chloroplast genome sequences in *Solanum tuberosum* accessions with T-type cytoplasm and in *Solanum tarijense* (*S. berthaultii*), are consistent with the idea that this species could be the maternal form for *S. tuberosum* Group Tuberosum.

All T-type plastomes of *S. tuberosum* had the same length, 155,296 bp, with a single exception. The genome of *Solanum tuberosum* L. cv. Desiree DQ231562 had a length of 155,312 bp. At the same time, another sequenced plastome of cv. Desiree NC_008096 had the same length as the other T-type plastomes. It is worth noting that the plastome of cv. Desiree DQ231562 was the first chloroplast genome of *Solanum tuberosum* L. sequenced, and may contain more errors due to imperfections in the technology. Thus, it is likely that its length may not be determined correctly. In addition, the level of similarity of this plastome with other *Solanum tuberosum* L. T-type plastomes is the lowest, which may also be a consequence of sequencing errors. The other three haplotypes identified for *Solanum tuberosum* differed insignificantly from each other. Hyplotype 4 differs by C instead of A at the 67,200th position in the genome, h2 differs by C instead of G at the 124913th position.

Previously, a low diversity of chloroplast genome sequences in European *S. tuberosum* cultivars with T-type cytoplasm was revealed using cpSSR markers. Furthermore, 151 samples with T-type cytoplasm out of 178 analyzed had identical haplotypes [31]. Based on this, it was assumed that the low sequence diversity of the chloroplast genome of *S. tuberosum* with T-type cytoplasm and the predominance of a single haplotype in the gene pool of modern cultivars may be the result of the widespread use of the imported US cultivar Rough Purple Chili as a female parent during the late blight epidemic of the 1840s [30,31]. However, the Vitelotte variety included in our study has been known since the early 19th century. (<1815, according to Potato Pedigree Database, https://www.plantbreeding.wur.nl/PotatoPedigree/pedigree_imagemap.php?id=17289, accessed on 31 October 2023) [52]. Thus, it is one of the first European varieties with a T-type cytoplasm that arrived in Europe before the *Phytophthora* epidemic. Its plastome does not differ from those of the modern potato varieties Desiree and Atlantic, and is identical to that of the diploid clone *S. tuberosum* and *Solanum berthaultii*.

Given the records on the origin of *S. tuberosum* Group Tuberosum from crossing the diploid species *S. tarijense* with T-type chloroplast DNA as a female with 4x Andigena, it can be assumed that the diversity of the T-genome in *S. tuberosum* Group Tuberosum was initially narrow and was not lost later as a result of the wide use of cultivar Rough Purple Chili in breeding. This could be related to the fact that a bottle neck had already occurred during emergence of the Chilean clade, including *S. tuberosum* cultivars (group Tuberosum) and their Chilean landrace progenitors (group Chilotanum), due to their origin from single or few interspecific crosses with the formation of the tetraploid genotype. At the same time, the relatively recent origin of *S. tuberosum* Group Tuberosum, according to the obtained dating, did not allow further formation of the diversity of chloroplast genome haplotypes due to divergence.

It should be noted that plastomes that belong to fertile cytoplasm types (A, P, M) belong to the D clade, while all sterilizing cytoplasm types of S. tuberosum (W, D, T) belong to clade E. Usually, the occurrence of pollen fertility problems in potatoes with D and W cytoplasm types is attributed to the introduction of chloroplast genomes of Mexican species in *S. tuberosum* Group Tuberosum. However, the T-type cytoplasm, which is characteristic of Chilean *S. tuberosum* Group Tuberosum as a whole, is also associated with pollen fertility problems. Meanwhile, as stated above, recent data suggest that the tetraploid nuclear genome of *S. tuberosum* Group Tuberosum was generated through hybridization between *S. tarijense* and Andean landraces [7,51]. These species are members of the E and D clades, respectively, which diverged about 4.7 MYA (CI95 = 3.78–5.70). Thus, the formation of CMS genotypes of *S. tuberosum* Group Tuberosum can apparently be explained by the so-called conflict between genes of the nuclear genome of D-clade species within the tetraploid genome of *S. tuberosum* Group Tuberosum and various types of cytoplasmic genomes in the E clade.

Due to the availability of data on the association of the cytoplasm type with the economically important traits of potatoes, the polymorphism of the amino acid sequences of proteins encoded by genes of the chloroplast genome was analyzed in six accessions sequenced in this study. As a result, differences between A and T genome types were identified for 15 genes, and differences between (A + P) and (T + W + D) genome groups were shown for 13 genes. However, positive selection (adaptive evolution) and a Ka/Ks ratio > 1 were observed only for *rbcL*. The chloroplast *rbcL* gene encodes the large subunit of Rubisco complex. In its protein sequence seven amino acid substitutions were found between groups of potato samples with the M, A, and P types of cytoplasm and a group of samples with the T, W, and D types of cytoplasm at positions 117, 142, 230, 262. 309, 439, and 449. Notably, that the amino acid at position 230 is included in the interface between the small subunit S1 and the large subunit B and it is involved in the formation of hydrogen bonds between the subunits [37]. Typically, most of the interface surfaces are hydrophobic. It is likely that the replacement of hydrophilic polar neutral Threonine with hydrophobic non-polar aliphatic Alanine may affect the stability of the interaction between the subunits of the Rubisco complex. The amino acid at position 262 is located in one of the four hydrophobic cores in the L subunit (second hydrophobic core of the C-terminal Domain) [37]. In this case, hydrophobic non-polar aliphatic Valine in the (M + A + P) group is replaced with hydrophilic polar neutral Threonine in (T + W + D) group. The amino acid at position 309 is included in the interface between the C-terminal domains of the two subunits in the L2 dimer [37]. However, in this case, Isoleucine is replaced by Methionine. Both of these amino acids are hydrophobic non-polar aliphatic, and it is unlikely that this kind of substitution can significantly affect the protein properties. The replacement of cysteine with alanine at position 449 also deserves attention. Cysteine is known to be involved in the formation of disulfide bonds, which affects protein folding and stability.

The chloroplast-localized Rubisco is the primary enzyme responsible for photosynthesis. The enzyme catalyzes the first step in net photosynthetic CO_2_ assimilation and photorespiratory carbon oxidation. It is known that Rubisco is inefficient as a catalyst for the carboxylation of ribulose-1,5-bisphosphate (RuBP) and is subject to competitive inhibition by O_2_, inactivation by loss of carbamylation, and dead-end inhibition by RuBP. These features make the Rubisco rate limiting for photosynthesis and a perspective target for increasing agricultural productivity [53].

It was previously shown that the evolution of *rbcL* could play an important role in adaptive radiation of some gymnosperm and angiosperm plants [54,55]. Thus, the revealed variations in the sequence of the *rbcL* gene between species with A and T-type cytoplasm may be one of the factors causing differences in economically important traits, especially in productivity (yield of tubers and their number).

## 4. Materials and Methods

### 4.1. Plant Material

Potato accessions with different types of cytoplasmic genomes from the collection of Russian Potato Research Centre were used for the study (Table 1). The cytoplasmic genome types were identified via the PCR method using a system of five molecular markers (A, T, SAC, D, and S) [1].

### 4.2. DNA Extraction and Sequencing

To isolate DNA preparations enriched with mitochondrial and chloroplast genome sequences, 5 g of fresh leaves sampled from individual plants were homogenized in 20 mL of STE buffer (0.4 M sucrose, 50 mM Tris (pH 7.8), 4 M EDTA-Na_2_, 0.2% BSA, 0.2% 2-mercaptoethanol). The resulting homogenate was filtered through two layers of Miracloth and two layers of Cheesecloth. The resulting filtrate was centrifuged at 3700× *g* for 20 min at 0 °C, and then the supernatant was collected into new tubes and centrifuged at 18,000× *g* at 0 °C, after which the obtained precipitate was used for DNA extraction. DNA was extracted with the Quick-DNA Plant/Seed Kit (Zymo Research, Irvine, CA, USA). The concentration of DNA samples was quantified using the QuDye HS kit (Lumiprobe RUS Ltd., Moscow, Russia) on a Qubit 4 fluorimeter (Thermo Fisher Scientific, Waltham, MA, USA). The libraries for chloroplast genome sequencing were prepared using the NEBNext^®^ Ultra™ II FS DNA Library Prep Kit (New England Biolabs Ltd., Ipswich, MA, USA) according to the manufacturer’s protocol. The obtained libraries were sequenced on the Illumina NovaSeq 6000 with a read length of 150 PE.

### 4.3. Chloroplast Genome Assembly and Annotation

To evaluate the quality of raw sequencing data, FastQC (version 0.11.9) [56] and MultiQC [57] (version 1.10.1) software were applied. Adapters were trimmed with Skewer (version 0.2.2) [58]. The trimmed reads number varied among samples from 1,887,454 to 8,790,748 (Table 1). Assembly of plastid genomes was performed with the GetOrganelle software (version 1.7.6.1) [59] with the following settings “-R 30 -k 21,31,41,51,61,71,81,91,101,111,117 -F embplant_pt -J 1 -M 1 -w 65”, and using *S. tuberosum* cultivar Colomba chloroplast complete genome as a seed sequence (MZ030723.1). All assembled plastomes had a high sequencing depth (Table 1). Genome annotation based on sequence similarity with the complete chloroplast genome sequence of *S*. *stenotomum* subsp. *goniocalyx* isolate GON1 (MT120855) was performed in Geneious software (version 2023.0.2) [60]. GeSeq [61], by means of ARAGORN program [62] with the default settings, was applied to detect tRNA genes. To manually verify the genomes annotation, all annotated protein-coding sequences (CDS) were extracted and translated into a set of amino acid sequences, and then aligned with the MAFFT plugin of Geneious tool [63]. Annotated plastid genome sequences were submitted to GenBank (Table 2). Circular genome maps were drawn using the CHLOROPLOT web server [64].

### 4.4. Simple Sequence Repeats Analysis

Simple sequence repeats were detected and located in the chloroplast genomes with the GMATA tool (version 2.3) [65]. For the cp SSR identification, the following minimal number of repeated units were applied: 10 for mononucleotide repeats; 5 for dinucleotide repeats; 4 for trinucleotide repeats; and 3 for tetra-, penta-, and hexanucleotide repeats. The position of SSRs relative to CDS sequences were identified using bedtools [66].

### 4.5. Analysis of Repetitive Elements

The search for repeating sequences was performed using the online version of the REPuter [67] software with the following parameters: the minimum repeat length of 20 bp and the hamming distance equal to 0. Every detected repetitive sequence (except for the inverted repeat consisting of IRb and IRa subunits) was analyzed. Each identified repeat was observed at least twice in the analyzed genome. In case all the subunits of a particular repeat were nested in those of another longer repeat, the former was excluded from the consideration. The position of repetitive elements relative to gene sequences were identified using bedtools [66]. The plots were created using the ggplot2 [68] package and lumina [69] palette in R v.4.3.1 [70].

### 4.6. Genome Comparison and Nucleotide Diversity Analysis

Alignment of complete sequences of chloroplast genomes of six *Solanum tuberosum* accessions was accomplished in the MAFFT plugin in the Geneious tool, and pairwise similarity indexes were estimated [60]. Nucleotide diversity( Pi) was estimated in DnaSP v6 with Sliding window option with window length = 500 and step size = 100 [71].

### 4.7. Phylogenetic Reconstruction

The second IR region was removed from the multiple sequence alignment. A tree of the six sequenced plastomes was reconstructed with RAxML version 8.2.11, using the GTR CAT model [72]. To analyze the phylogenetic relationships of T-, D-, W-, P-, and A-type plastomes, they were aligned with sequences of *Solanum* and *Jaltomata* species from the GenBank database available on January 2023 (Table S4). Phylogenetic reconstruction was performed in FastTree 2.1.11+galaxy1 [73,74,75].

### 4.8. Molecular Dating Analyses

Time of divergence was estimated using RelTime-ML implemented in MEGA11 and MEGA-CC [76,77,78]. The RelTime approach is based on a relative rate framework and performs dating analyses by relaxing the assumption of a strict molecular clock in a phylogeny [79]. The following options were applied to the analysis: General Time Reversible model, Rates among Sites = Gamma Distributed, No of Discrete Gamma Categories = 5, Gaps/Missing Data Treatment = Use all sites. The following divergence time estimates have been used for dating from the study of T. Särkinen et al.: Thelopodium/other Solanum—15.5 MYA, *S. melongena*—*S. tuberosum*—14.3 MYA, *S. melongena*—*S. macrocarpon*—3.4 MYA, *S. tuberosum*—*S. piurae*—7.1 MYA, *S. lycopersicon*—*S. peruvianum*—2.0 MYA, *S. lycopersicon*—*S. tuberosum*—8.0 MYA [36]. Phylogenetic trees with estimated divergence times were visualized in FigTree v1.4.4 (http://tree.bio.ed.ac.uk/software/figtree/, accessed on 31 October 2023) [80].

### 4.9. Adaptive Evolution Analysis

The amino acid sequence polymorphism of proteins encoded by chloroplast genome genes was analyzed in six samples sequenced in this study. The nonsynonymous (Ka) and synonymous (Ks) substitution rates and Ka/Ks ratio were also evaluated using Mega 11 software for each of the genes in which polymorphism was detected [76].

## 5. Conclusions

For the first time, a comparative analysis of sequences of potato plastomes with different cytoplasm types has been carried out and their origin times have been estimated. As a result, the presence of two main groups of chloroplast genomes among cultivated potato was confirmed. The first group includes W, D, T-, and the second group consists of M, A and P- cytoplasm types, respectively.

Based on the phylogenetic analysis of the complete plastome sequences, five main evolutionary branches of chloroplast genomes (clades A–E) can be distinguished within the Petota section. Clade D comprises accessions with M-, A, and P types of cytoplasm. Samples with A- and P- cytoplasm formed isolated distant groups within a large and polymorphic group of samples with M-type cytoplasm, suggesting that the A and P genomes arose independently. Moreover, given the independent origin of the A- and P-type cytoplasmic groups, it seems unlikely that Andean cultivated tetraploids (S. tuberosum group Andigena) evolved directly from early landrace diploids (S. tuberosum groups Stenotomum and Phureja) through autopolyploidy. Accessions with the sterilizing cytoplasm types W, D, T are part of the E clade. The divergence time between the D and E clades can be estimated as 4.71 MYA (3.78–5.70). The M type of cytoplasm appears to be the most ancient.

The findings suggest that the diversity of the T-genome in *S. tuberosum* Group Tuberosum could be initially narrow due to bottle neck already at the origin of the Chilean clade.

Revealed variations in the *rbcL* gene sequence may be one of the factors causing differences in appearance of economically important traits between species with A and T-type cytoplasm.

## Figures and Tables

**Table 1:** General features of sequenced chloroplast genomes.

Accessions	Type of Cytoplasm	Genbank Accession Number	Read Pairs Number	Average Genome Coverage	Sequence Length, bp			
					IR	LSC	SSC	Total
*S. tuberosum* Group Tuberosum cv. Nakra	W	OR632697	3,302,337	784×	25,592	85,991	18,374	155,549
*S. tuberosum* Group Tuberosum cv. Vitelotte	T	OR632698	7,906,999	761×	25,593	85,737	18,373	155,296
*S. tuberosum* Group Tuberosum cv. Barin	D	OR632702	8,790,748	697×	25,593	86,003	18,373	155,562
*S. tuberosum* Group Phureja	P	OR632699	5,036,719	804×	25,593	85,930	18,376	155,492
*S. tuberosum* Group Andigenum a3	A	OR632700	1,887,454	531×	25,593	85,968	18,364	155,518
*S. tuberosum* Group Andigenum a4	A	OR632701	5,751,620	778×	25,593	85,967	18,364	155,517

IR—inverted repeat, LSC—large single copy, SSC—small single copy.

**Table 2:** Localization of molecular markers for identification of cytoplasm types in potato chloroplast genome. Marker names are provided according to K. Hosaka and R. Sanetomo [1].

Marker	Primer Sequence	References	Position in Chloroplast Genome of *S*. *tuberosum* cv. Barin with D Type of Cytoplasm
T	GGAGGGGTTTTTCTTGGTTG	[27]	52,459–52,478
	AAGTTTACTCACGGCAATCG		52,882–52,901
S	GGTTCGAATCCTTCCGTC	[31]	7282–7299
	GATTCTTTCGCATCTCGATTC		GATTCTTTCGC/TATCTCGATTC 7126–7146
SAC	TTGGAGTTGTTGCGAATGAG	[1]	63,681–63,700
	GTTCCCTAGCCACGATTCTG		63,973–63,992
D	CGGGAGGTGGTGTACTTTCT	[32]	not found
	ACGGCTGACTGTGTGTTTGA		not found
A	AACTTTTTGAACTCTATTCCTTAATTG	[1]	115,410–115,436
	ACGCTTCATTAGCCCATACC		116,577–116,596

**Table 3:** Polymorphism of plastome-encoded protein sequences in *S. tuberosum* accessions with different cytoplasm types.

Function	Gene	Length, bp	Substitutions Number	Alignment Position	Cytoplasm Type					A/T Genomes Substitution	(A + P)/(T + W + D) Genomes Substitution
					P	A	D	W	T		
ATP synthase	*atpB*	498	1	99	S	S	R	R	R	S/R	S/R
Cytochrome c biogenesis protein	*ccsA*	313	1	199	K	K	Q	Q	K		
Maturase K	*matK*	509	3	54	F	F	L	L	L	F/L	F/L
				191	L	L	W	W	L		
				333	D	D	N	N	D		
NADH dehydrogenase	*ndhA*	363	1	31	I	I	V	V	V	I/V	I/V
	*ndhB*	510	1	70	M	M	M	M	L	M/L	
	*ndhF*	739	3	466	G	G	G	G	D	G/D	
				649	L	L	I	L	L		
				677	L	L	F	F	L		
	*ndhG*	176	1	44	V	V	I	I	I	V/I	V/I
Photosystem II	*psbC*	461	1	425	L	L	F	F	F	L/F	L/F
Rubisco large subunit	*rbcL*	477	7	117	F	F	L	L	L	F/L	F/L
				142	T	T	V	V	V	T/V	T/V
				230	T	T	A	A	A	T/A	T/A
				262	V	V	T	T	T	V/T	V/T
				309	I	I	M	M	M	I/M	I/M
				439	Q	Q	R	R	R	Q/R	Q/R
				449	C	C	A	A	A	C/A	C/A
DNA dependent RNA polymerase	*rpoB*	1070	2	31	V	V	L	L	L	V/L	V/L
				587	I	I	V	V	I		
	*rpoC1*	688	1	668	V	V	A	A	A	V/A	V/A
	*rpoC2*	1392	1	1052	F	F	V	V	V	F/V	F/V
Large subunit of ribosome	*rpl14*	122	1	57	I	I	V	V	V	I/V	I/V
	*rpl20*	128	1	65	I	I	L	L	I		
Small subunit of ribosome	*rps11*	138	1	77	A	A	V	V	A		
	*rps15*	87	2	13	E	E	K	K	K	E/K	E/K
				80	E	E	D	D	D	E/D	E/D
	*rps3*	218	2	103	L	L	F	F	F	L/F	L/F
				217	E	E	A	A	E		
	*rps4*	201	1	39	V	G	G	G	G		
Proteins of unknown function	*ycf1*	1887	10	360	F	F	L	L	L	F/L	F/L
				487	T	T	K	K	K	T/K	T/K
				581	K	K	E	E	E	K/E	K/E
				972	F	F	L	L	L	F/L	F/L
				1008	I	I	L	L	L	I/L	I/L
				1116	I	I	K	K	K	I/K	I/K
				1258	D	D	N	N	D		
				1319	Q	Q	Q	Q	K	Q/K	
				1321	K	K	Q	Q	K		
				1345	S	L	S	S	S	L/S	
	*ycf2*	2278	1	606	Y	Y	S	S	Y		
										29	25

**Table 4:** The non-synonymous (Ka) and synonymous (Ks) substitution rates and Ka/Ks ratio for 20 genes in a group of six potato accessions.

Gene	Ka	Ks	Ka/Ks
*atpB*	0.0005	0.0009	0.55556
*ccsA*	0.0007	0.0025	0.28
*matK*	0.0014	0.0015	0.93333
*ndhA*	0.0007	0.0044	0.15909
*ndhB*	0.0003	0	NA
*ndhF*	0.0007	0.0029	0.24138
*ndhG*	0.0015	0.0047	0.31915
*psbC*	0.0006	0	NA
*rbcL*	0.0056	0.001	5.6
*rpl15*	0.0022	0.0038	0.57895
*rpl20*	0.0018	0	NA
*rpoB*	0.0005	0.0023	0.21739
*rpoC1*	0.0004	0.0019	0.21053
*rpoC2*	0.0002	0.001	0.2
*rps3*	0.0022	0.0064	0.34375
*rps4*	0.0007	0	NA
*rps11*	0.0018	0	NA
*rps15*	0.006	0.0093	0.64516
*ycf1*	0.0016	0.0042	0.38095
*ycf2*	0.0001	0.0004	0.25

**Table 5:** Amino acid substitutions in Rubisco large subunits of *S. tuberosum* accessions with different cytoplasm types.

Codon No	Amino Acid Changes		Type of Changes ^a^	Location of Residue ^b^
	M + A + P	T + W + D		
117	F	L	HR => HN	-helix C
142	T	V	UP => HN	-helix D
230	T	A	UP => HN	-helix 2
262	V	T	HN => UP	between helix 3 и-strand 4
309	I	M	HN => HN	-strand F
439	Q	R	UP => UB	-helix G
449	C	A	UP => HN	-helix G

a—Side chain type changes. Types abbreviations: A–acidic (negatively charged); B–basic (positively charged); H–hydrophobic; N–nonpolar aliphatic; P–polar uncharged; R–aromatic; U–hydrophilic [38]. b—Location of residue according to [37].

**Table 6:** Haplotypes of chloroplast genomes of *Solanum* accessions with T-type cytoplasm.

GenBank No	Lengh, bp	Haplotype	Sample
DQ231562	155312	h1	*Solanum tuberosum* L. cv. Desiree
MH021416	155296	h2	*Solanum berthaultii* voucher PI 498105
MT511702	155296	h2	*Solanum tuberosum* chloroplast clone 7506-01 (2n)
MW307947	155296	h2	*Solanum tuberosum* cultivar Atlantic
MZ030720	155296	h2	*Solanum tuberosum* cultivar Atlantic
NC_008096	155296	h2	*Solanum tuberosum* cv. Desiree
OR632698	155296	h2	*Solanum tuberosum* cv. Vitelotte (this study)
MT511703	155296	h3	*Solanum tuberosum* chloroplast clone 08675-21 (2n)
MT511708	155296	h3	*Solanum tuberosum* chloroplast clone DW84-1457 (2n)
MT511709	155296	h3	*Solanum tuberosum* chloroplast clone H412-1 (2n)
MW307946	155296	h3	*Solanum tuberosum* cultivar Yanshu No. 4
MW307948	155296	h3	*Solanum tuberosum* cultivar Favorita
MW307949	155296	h3	*Solanum tuberosum* cultivar Shepody
MZ030723	155296	h3	*Solanum tuberosum* cultivar Colomba
MZ030724	155296	h3	*Solanum tuberosum* cultivar Spunta
KM489056	155296	h4	*Solanum tuberosum*
MH021575	155297	h5	*Solanum tarijense*
MH021576	155295	h6	*Solanum tarijense*
OM638071	155295	h6	*Solanum tarijense*
MH021573	155299	h7	*Solanum tarijense*

## Data Availability

The sequenced and annotated potato plastomes have been uploaded to NCBI’s GenBank (OR632697, OR632698, OR632699, OR632700, OR632701, OR632702).

## Supplementary Materials

The following supporting information can be downloaded at: https://www.mdpi.com/article/10.3390/plants12233995/s1, Table S1. Comparison of microsatellites in the chloroplast genomes of Solanum tuberosum accessions; Table S2. Repetitive element content in plastomes of S. tuberosum accessions; Table S3. Percentage of pairwise similarity between *S. tuberosum* plastomes; Table S4. Plastome sequences of *Solanum* and *Jaltomata* species from the GenBank database; Figure S1. Physical maps of the chloroplast genomes of *S. tuberosum* (A) Group Tuberosum with cytoplasmic type T, (B) Group Phureja with cytoplasmic type P, (C) Group Andigenum 3a with cytoplasmic type A, (D) Group Andigenum a4 with cytoplasmic type A, (E) Group Tuberosum with cytoplasmic type D, (F) Group Tuberosum with cytoplasmic type W.; Figure S2. Unrooted phylogenetic tree of *Solanum* and *Jaltomata* plastomes.
