# Supplementary material for: Comparative Study of Plastomes in Solanum tuberosum with Different Cytoplasm Types
Source: Plants (Basel). 2023 Nov 28;12(23):3995. doi: 10.3390/plants12233995 (PMC10708428; doi:10.3390/plants12233995)
Supplement: Supplementary file 1 [file plants-12-03995-s001.zip › plants-2720377-supplementary-Table S1.pdf]

Table S1. Comparison of microsatellites in the chloroplast genomes of *Solanum tuberosum* accessions

**Accession**	**Plastome Length, bp**	**Total SSR Number**	**Total Repeats Length, bp**	**% of Plastome Length**	**SSR Number in CDS regions**	**Total SSR Number per type**					
						**Mono**	**Di**	**Tri**	**Tetra**	**Penta**	**Hexa**
S. tuberosum Group Tuberosum cv. Nakra W	155549	56	625	0.4018	6	39	6	2	8	1	0
S. tuberosum Group Tuberosum cv. Vitelotte T	155296	53	588	0.3786	6	36	6	2	8	1	0
S. tuberosum Group Phureja P	155492	51	584	0.3756	6	34	6	2	8	1	0
S. tuberosum Group Andigenum a3A	155518	53	588	0.3781	6	36	6	2	8	1	0
S. tuberosum Group Andigenum a4A	155517	53	588	0.3781	6	36	6	2	8	1	0
S. tuberosum Group Tuberosum cv. Barin D	155562	55	616	0.3960	6	38	6	2	8	1	0
