# Supplementary material for: Comparative Study of Plastomes in Solanum tuberosum with Different Cytoplasm Types
Source: Plants (Basel). 2023 Nov 28;12(23):3995. doi: 10.3390/plants12233995 (PMC10708428; doi:10.3390/plants12233995)
Supplement: Supplementary file 1 [file plants-12-03995-s001.zip › plants-2720377-supplementary-Table S2.pdf]

Table S2. Repetitive element content in plastomes of *S. tuberosum* accessions

**Species**	**Plastome Length, bp**	**Total Repeats Number**	**Total Repeats Length, bp**	**% of Plastome Length**	**Number of repeats with all subunits inside gene sequences**	**Number of repeats with all subunits outside gene sequences**
S. tuberosum Group Tuberosum **W**	155549	11	628	0,4037	4	4
S. tuberosum Group Tuberosum **T**	155296	12	685	0,4411	5	4
S. tuberosum Group Phureja **P**	155492	12	685	0,4405	5	4
S. tuberosum Group Andigenum a3 **A**	155518	13	727	0,4675	5	4
S. tuberosum Group Andigenum a4 **A**	155517	13	727	0,4675	5	4
S. tuberosum Group Tuberosum **D**	155562	11	658	0,423	4	4
