# Supplementary material for: Comparative Study of Plastomes in Solanum tuberosum with Different Cytoplasm Types
Source: Plants (Basel). 2023 Nov 28;12(23):3995. doi: 10.3390/plants12233995 (PMC10708428; doi:10.3390/plants12233995)
Supplement: Supplementary file 1 [file plants-12-03995-s001.zip › plants-2720377-supplementary-Table S3.pdf]

Table S3. Percent of pairwise similiarity between *S. tuberosum* plastomes

	S. tuberosum Group Tuberosum cv. Nakra W	*S. tuberosum* Group Tuberosum cv. Barin D	*S. tuberosum* Group Tuberosum cv. Vitelotte T	*S. tuberosum* Group Phureja P	*S. tuberosum* Group Andigenum a3 A	*S. tuberosum* Group Andigenum a4 A
S. tuberosum Group Tuberosum cv. Nakra W		99.932	99.692	99.764	99.794	99.795
S. tuberosum Group Tuberosum cv. Barin D	99.932		99.634	99.708	99.74	99.741
S. tuberosum Group Tuberosum cv. Vitelotte T	99.692	99.634		99.583	99.614	99.614
S. tuberosum Group Phureja P	99.764	99.708	99.583		99.903	99.904
S. tuberosum Group Andigenum a3 A	99.794	99.74	99.614	99.903		99.999
S. tuberosum Group Andigenum a4 A	99.795	99.741	99.614	99.904	99.999	
