# Supplementary figures and images for: Comparative Study of Plastomes in Solanum tuberosum with Different Cytoplasm Types
Source: Plants (Basel). 2023 Nov 28;12(23):3995. doi: 10.3390/plants12233995 (PMC10708428; doi:10.3390/plants12233995)

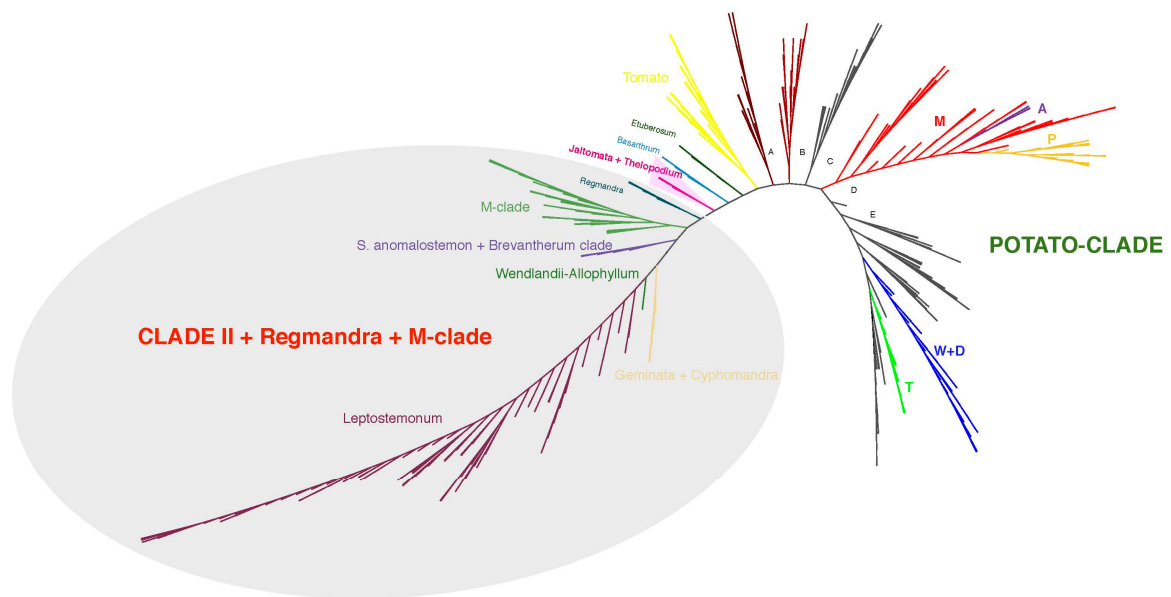

Figure S2. Unrooted phylogenetic tree of *Solanum* and *Jaltomata* plastomes.

Supplement: Supplementary file 1 [file plants-12-03995-s001.zip › plants-2720377-supplementary-Figure S2.pdf]
