# Supplementary material for: Comparative Study of Plastomes in Solanum tuberosum with Different Cytoplasm Types
Source: Plants (Basel). 2023 Nov 28;12(23):3995. doi: 10.3390/plants12233995 (PMC10708428; doi:10.3390/plants12233995)
Supplement: Supplementary file 1 [file plants-2720377-supplementary-Figure S1.pdf › plants-2720377-supplementary-Figure S1.pdf]

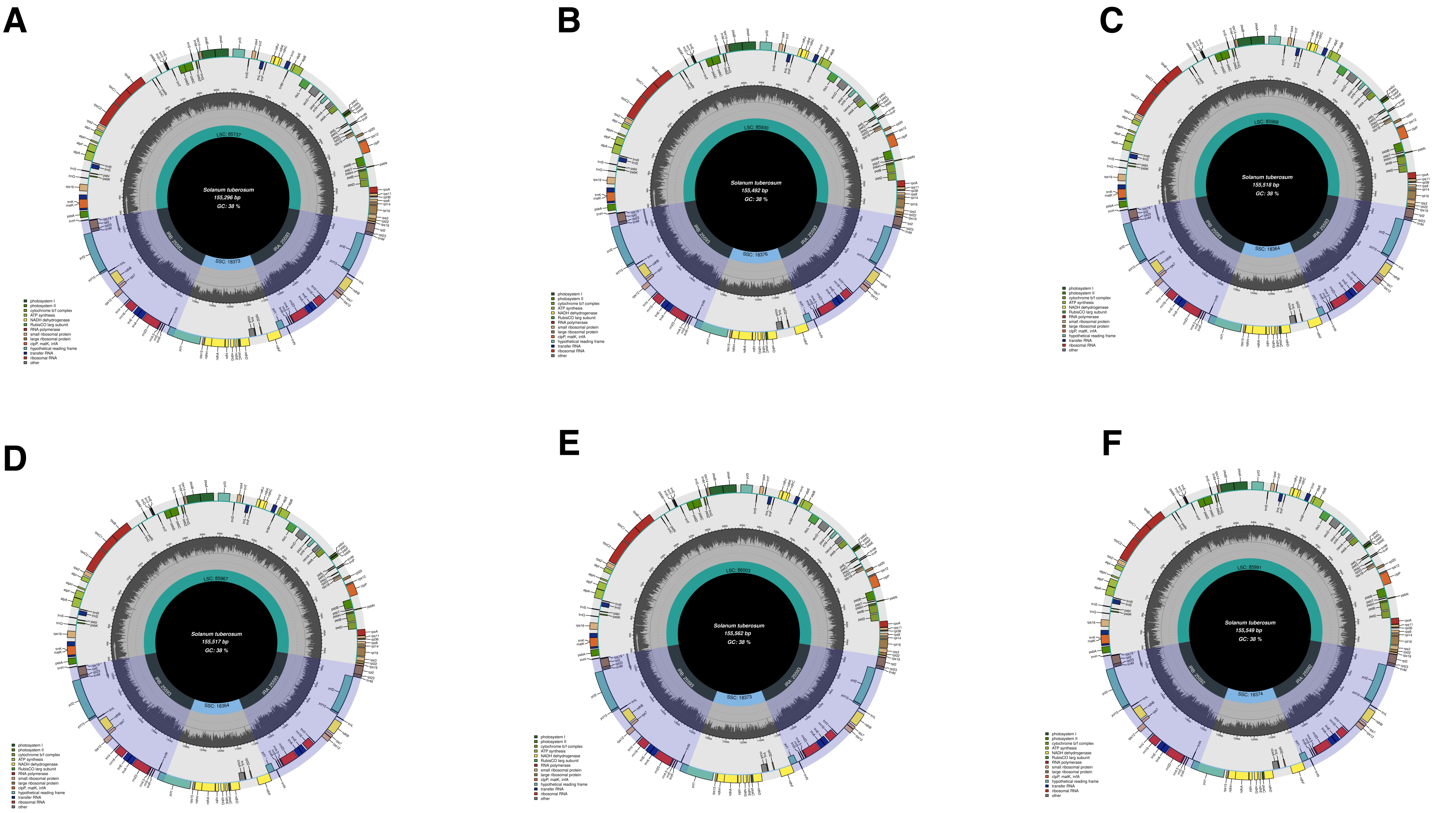

Figure S1. Physical maps of the chloroplast genomes of *S. tuberosum* (**A**) Group Tuberosum with cytoplasmic type T, (**B**) Group Phureja with cytoplasmic type P, (**C**) Group Andigenum 3a with cytoplasmic type A, (**D**) Group Andigenum a4 with cytoplasmic type A, (**E**) Group Tuberosum with cytoplasmic type D, (**F**) Group Tuberosum with cytoplasmic type W.
